# Auxilin Depletion Causes Self-Assembly of Clathrin into Membraneless Cages *In Vivo*

**DOI:** 10.1111/j.1600-0854.2008.00764.x

**Published:** 2008-06-11

**Authors:** Jennifer Hirst, Daniela A Sahlender, Sam Li, Nienke B Lubben, Georg H H Borner, Margaret S Robinson

**Affiliations:** 1 Cambridge Institute for Medical Research, University of Cambridge, Wellcome Trust/MRC Building, Addenbrooke’s HospitalHills Road, Cambridge CB2 0XY, UK; 2 Medical Research Council Laboratory of Molecular BiologyCambridge CB2 2QH, UK

**Keywords:** AP-1, AP-2, auxilin, clathrin, coated vesicle, GGA, intracellular membranes, plasma membrane, TGN

## Abstract

Auxilin is a cofactor for Hsc70-mediated uncoating of clathrin-coated vesicles (CCVs). However, small interfering RNA (siRNA) knockdown of the ubiquitous auxilin 2 in HeLa cells only moderately impairs clathrin-dependent trafficking. In this study, we show that HeLa cells also express auxilin 1, previously thought to be neuron specific, and that both auxilins need to be depleted for inhibition of clathrin-mediated endocytosis and intracellular sorting. Depleting both auxilins cause an ∼50% reduction in the number of clathrin-coated pits at the plasma membrane but enhances the association of clathrin and adaptors with intracellular membranes. CCV fractions isolated from auxilin-depleted cells have an ∼1.5-fold increase in clathrin content and more than fivefold increase in the amount of AP-2 adaptor complex and other endocytic machinery, with no concomitant increase in cargo. In addition, the structures isolated from auxilin-depleted cells are on average smaller than CCVs from control cells and are largely devoid of membrane, indicating that they are not CCVs but membraneless clathrin cages. Similar structures are observed by electron microscopy in intact auxilin-depleted HeLa cells. Together, these findings indicate that the two auxilins have overlapping functions and that they not only facilitate the uncoating of CCVs but also prevent the formation of nonproductive clathrin cages in the cytosol.

Cycles of coat assembly and disassembly are essential for clathrin-dependent trafficking. Formation of clathrin-coated vesicles (CCVs) requires the recruitment of cytosolic coat components onto membranes, where they assemble to form the coat. These coat components include clathrin, adaptors and various accessory proteins, such as dynamin, which is required for the fission event that yields the transport vesicle. Fusion of the CCV with its target membrane requires the disassembly of the coat, and results in the recycling of clathrin and other coat components back into the cytosol for further rounds of vesicle formation. It has been proposed that the ATP-dependent uncoating of CCVs is mediated by the molecular chaperone Hsc70, and its cofactors auxilin 1 and auxilin 2 (reviewed in 1). Auxilin 1 (DNAJC6) was first identified as a minor, tissue-specific clathrin assembly protein purified from bovine brain (2). A second, ubiquitously expressed isoform of auxilin, auxilin 2 (also known as cyclin G-associated kinase), was subsequently discovered. Auxilin 2 shares 50% sequence identity with the neuronal-specific auxilin 1 (3–5), but it has an additional serine/threonine kinase domain.

Both auxilins bind clathrin, adaptors (5–8) and dynamin (9). Their C-terminal J domain confers the specificity for binding to Hsc70, the uncoating ATPase (adenosine triphosphatase) (10). The proposed mechanism is that auxilin binds to forming clathrin-coated buds shortly before fission, where it specifically recruits ATP-bound Hsc70. Auxilin’s J domain stimulates the ATPase activity of Hsc70, which in turn stabilizes Hsc70’s association with clathrin. This interaction ultimately drives the dissociation of the clathrin coat into triskelia and hence the uncoating of CCVs (11,12).

The requirement for auxilin as well as Hsc70 in disassembling clathrin from CCVs has been demonstrated in a number of studies and was a crucial step in establishing a physiological role for Hsc70 in the uncoating reaction. These studies were performed in a variety of organisms, including yeast, *Caenorhabditis elegans* and Drosophila, all of which express only one isoform of auxilin. Deletion of the auxilin gene in Drosophila is lethal, while mutants with reduced auxilin function show genetic interactions with Hsc70 and clathrin (13). When auxilin is depleted in *C. elegans*, endocytosis of yolk protein in oocytes is markedly reduced, most of the worms arrest during larval development and clathrin dynamics are altered (14). Disruption of the yeast auxilin gene results in an accumulation of CCVs, impaired cargo delivery and slow cell growth (15,16). Auxilin and its interaction with Hsc70 have also been shown to be critical for clathrin-mediated endocytosis in the squid giant synapse (17). In addition to its role as a cochaperone for Hsc70 in the uncoating of CCVs, auxilin has been proposed to have functions in the recruitment of clathrin and adaptors to membranes (18), in the exchange of clathrin during pit remodeling (19), and most recently in the scission of clathrin-coated pits (CCPs) to form free vesicles through its ability to bind dynamin (20).

In spite of evidence from numerous systems that auxilin plays a key role in CCV dynamics, studies in mammalian cells have produced contradictory results. Knockdown of the ubiquitous auxilin 2 in HeLa cells causes only relatively mild phenotypes. Internalization of both EGF and transferrin is moderately impaired (18,21), and trafficking of the cation-independent mannose 6-phosphate receptor (CIMPR) (18) and its ligand cathepsin D (7) is also partially affected. However, no accumulation of CCVs has been observed. In addition, there is conflicting data on the effects of auxilin 2 depletion on the recruitment of clathrin and adaptors (7,18,21) and on the exchange of clathrin between cytosol and membranes (18,21). Because all these data were obtained using transient RNA interference (RNAi) depletion of auxilin 2 in HeLa cells, it is difficult to integrate the various findings. The unexpectedly mild phenotypes also conflict with those seen in yeast, Drosophila and *C. elegans*(13–17).

A simple explanation for the discrepancy between the phenotype in mammalian cells and the phenotypes in other organisms is the existence of an alternative J-domain protein that might compensate for the absence of auxilin 2. Indeed, we recently reported that both auxilin 1 and auxilin 2 are present in CCVs prepared from HeLa cells (22). In this study, we resolve the conflicting literature demonstrating overlapping roles for auxilin 1 and auxilin 2 in clathrin-mediated endocytosis and intracellular trafficking, and in the dynamics of clathrin assembly/disassembly. These results give insights into how auxilin 1 and auxilin 2 may be functioning in the brain, where both isoforms are normally coexpressed, and also provide us with a useful tool for proteomic analysis of isolated CCVs.

## Results

### Auxilin 1 is expressed in numerous non-neuronal cell lines

**Figure 1:**
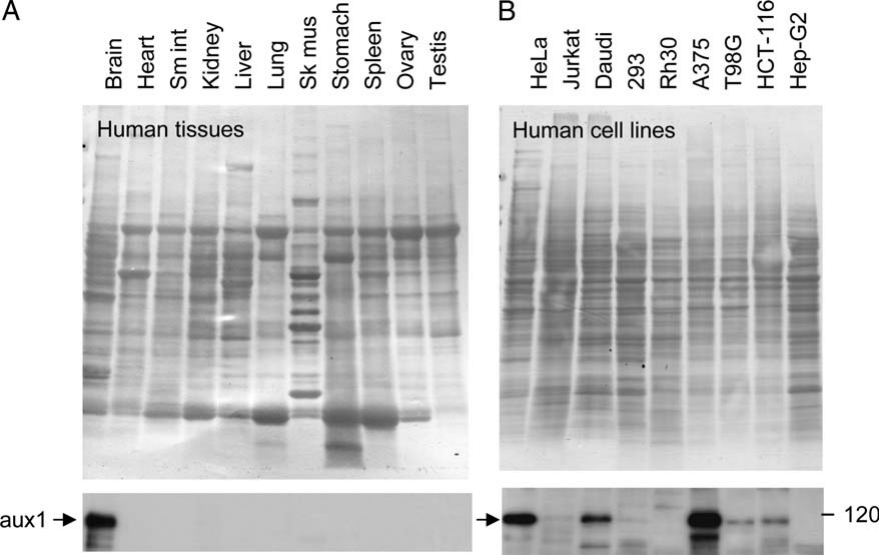
Auxilin 1 expression patterns Coomassie Blue-stained commercial blots of human tissues (A) and human cell lines (B) were probed with an antibody against auxilin 1. Sm int = small intestine; Sk mus = skeletal muscle.

Auxilin 1 was originally identified as a minor component of CCVs from bovine brain (2), and it has been reported to be a neuron-specific protein (for a review, see 1). Genechip data support the restricted expression of auxilin 1 to brain (SymCard Expression Profile for DNAJC6; http://symatlas.gnf.org/SymAtlas/), as does antibody labeling of a multiple tissue western blot (Figure 1A). However, in our recent study on the protein composition of CCVs from HeLa cells (a cervical carcinoma cell line), we found both auxilin 1 and auxilin 2 in our CCV fractions (22). Western blot analysis of a number of tissue culture cell lines reveals that several other non-neuronal cell lines also express the ‘neuron-specific’ auxilin 1 (Figure 1B). In particular, we found significant expression in Daudi (lymphoma) and A375 (amelanotic melanoma) cell lines and weak expression in T98G (glioma) and HCT-116 (colon epithelial carcinoma) cell lines. Thus, auxilin 1 expression is not restricted to brain-derived cells.

### Relative abundance of auxilins

To estimate the relative abundance of auxilin 1 and auxilin 2 in HeLa CCVs, we used our previously published proteomics data (22) to calculate exponentially modified Protein Abundance Indices (emPAIs; *Materials and Methods*). emPAI values are proportional to molar protein concentrations and thus can be used to estimate relative levels of auxilins. emPAI was determined at 0.145 and 0.197 for auxilin 1 and auxilin 2, respectively, equivalent to a molar ratio of auxilin 1/auxilin 2 ≈1:1.4. Hence, our data suggest that auxilin 1 and auxilin 2 are present in very similar amounts in HeLa cell CCVs.

### Auxilin depletion inhibits clathrin-mediated endocytosis

**Figure 2:**
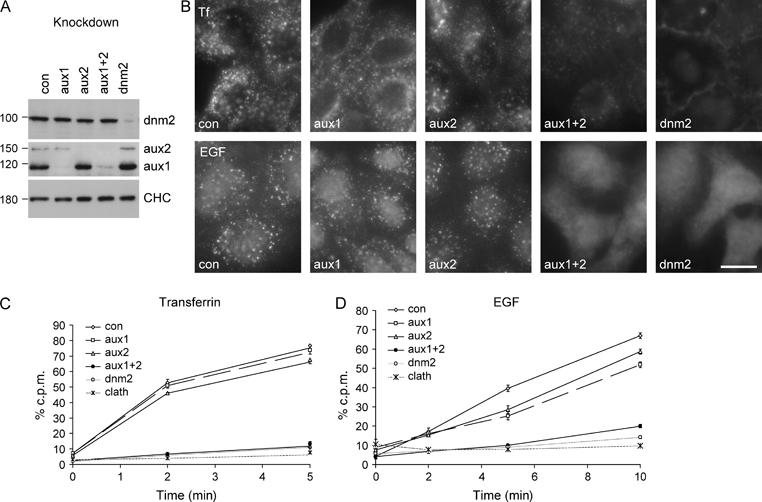
Auxilin depletion blocks clathrin-mediated endocytosis A) HeLa cells were depleted of auxilin 1, auxilin 2, both auxilins or dynamin by siRNA. Cell lysates were analyzed by western blotting; clathrin heavy chain (CHC) was used as a loading control. B) HeLa cells were depleted of auxilins or dynamin by siRNA and pulsed with 10 μg/mL Alexa Fluor 594-labeled transferrin or 400 ng/mL Alexa Fluor 488-labeled EGF for 10 min at 37°C. Scale bar: 20 μm. C and D) Cells were depleted of auxilins, dynamin or clathrin by siRNA, allowed to bind 500 nCi/mL (equivalent to ∼2 μg/mL for transferrin and ∼2 ng/mL for EGF) ^125^I-labeled transferrin or EGF at 4°C and then shifted to 37°C for the indicated length of time. Culture supernatants were harvested, ligand remaining at the cell surface was removed with an acid wash and cells were solubilized with NaOH. Radioactivity was quantified in all three fractions and expressed as a percentage of total counts per minute (c.p.m.). Only the cell-associated counts are shown here. Each point is derived from at least four separate experiments performed in triplicate, and the error bars show the standard error of the mean.

The function of auxilin 2 has been studied using RNAi depletion, mainly in HeLa cells, and only moderate phenotypes have been reported (7,18,21). We speculated that auxilin 1 might have partially compensated for auxilin 2 in these studies. To test this possibility, we used small interfering RNA (siRNA) to knock down the expression of both auxilin 1 and auxilin 2 in HeLa cells. For comparison, dynamin 2 was also knocked down. A 4-day protocol using two siRNA treatments efficiently depleted all three proteins by >90% (Figure 2A). To assess the effects of auxilin or dynamin depletion on clathrin-mediated endocytosis, we assayed the uptake of fluorescently labeled transferrin or EGF (Figure 2B). In control cells incubated with fluorescent ligand for 10 min at 37°C, both transferrin and EGF are internalized into punctate structures that resemble early endosomes. Similar uptake was observed in cells depleted of either auxilin 1 or auxilin 2 alone. However, there was an inhibition in the internalization of transferrin and EGF in cells depleted of both auxilins, similar to that seen in cells depleted of dynamin 2.

To quantify these effects, cells that had been depleted of auxilins or dynamin were incubated with radiolabeled ligand at 4°C to allow binding to occur and then shifted to 37°C for up to 10 min. There were only subtle reductions in the rates of uptake of transferrin (Figure 2C) or EGF (Figure 2D) in the auxilin 1 or auxilin 2 single knockdowns. However, in agreement with the fluorescence data, depletion of both auxilins or of dynamin resulted in an inhibition of transferrin and EGF uptake at early time-points, similar to that seen following clathrin depletion. Recovery of transferrin uptake was seen at longer times of incubation (results not shown). In addition, there was a twofold to threefold increase in the amount of transferrin bound to the plasma membrane in cells depleted of either dynamin or the two auxilins (Figure S1), suggesting an accumulation of transferrin receptor at the cell surface. These results demonstrate that depletion of both auxilins in HeLa cells is required for an inhibition of clathrin-mediated endocytosis and thus that auxilin 1 and auxilin 2 have overlapping functions.

### Effects of auxilin depletion on clathrin coats at the plasma membrane

**Figure 3:**
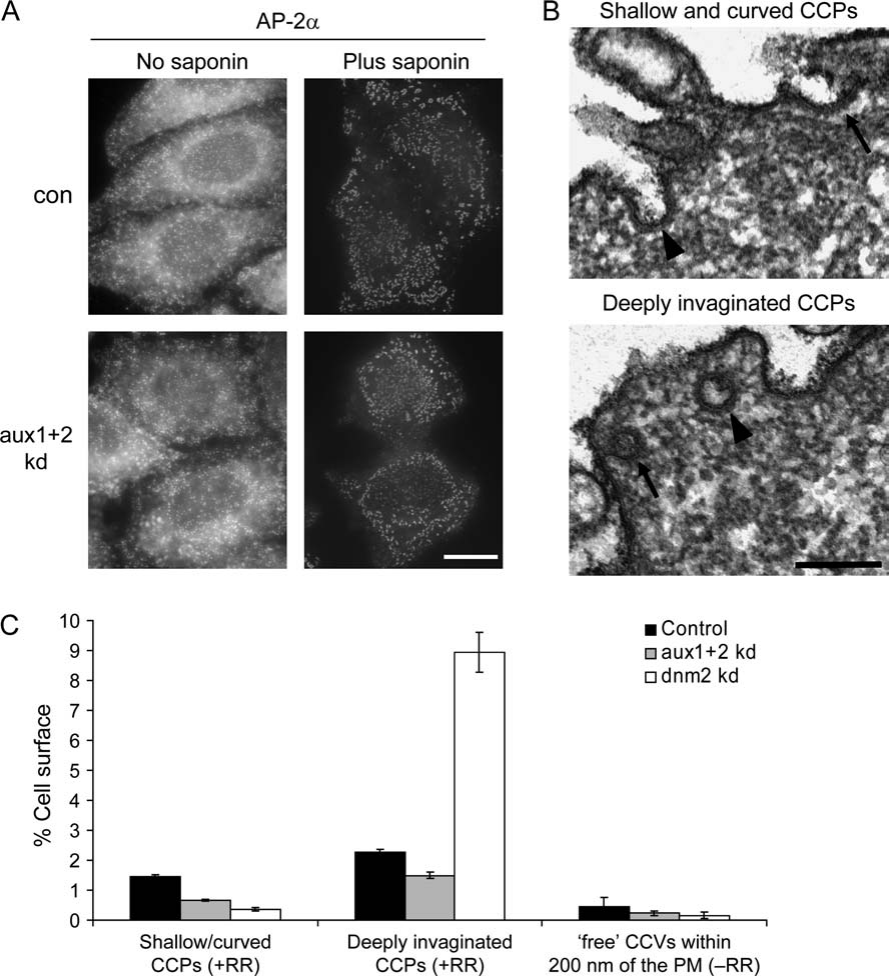
Auxilin depletion causes a reduction of CCPs at the plasma membrane A) Control cells or cells depleted of auxilin 1 and auxilin 2 were incubated with or without 0.05% saponin prior to fixation and labeled for AP-2α. Scale bar: 20 μm. B) Electron micrographs of cells depleted of auxilin 1 and auxilin 2. Cells were fixed in the presence of ruthenium red to distinguish budding CCPs, which are still connected to the plasma membrane, from pinched-off free CCVs. Note the electron-dense layer formed by the ruthenium red highlighting the continuity of the plasma membrane and clathrin-coated structures at different stages during pit formation. The upper image shows a fully opened pit (arrow) and a ‘U’-shaped pit (arrowhead), which are scored as shallow and curved pits, respectively. The lower image shows an ‘omega’-shaped pit with a narrow opening (arrow) and a cross-sectioned CCP containing ruthenium red but with no visible opening (arrowhead), both of which are grouped as deeply invaginated pits. Scale bar: 200 nm. C) Morphometric analysis of clathrin-coated profiles at the plasma membrane in control, auxilin-depleted and dynamin-depleted cells. The percentage of clathrin-coated profiles at the plasma membrane was determined, averaging the results from two independent experiments. Error bars show the standard error of the mean.

We next investigated the effect of auxilin 1/auxilin 2 knockdown on CCP formation at the plasma membrane. Depletion of both auxilins in HeLa cells resulted in a change in cell morphology with most cells appearing more rounded, which made it difficult to assess changes at the cell surface by immunofluorescence. With this caveat in mind, we did not observe any significant alteration in the localization of AP-2, clathrin or the AP-2 accessory proteins epsin and clathrin assembly lymphoid myeloid leukemia protein (CALM) (Figure 3A and unpublished observations) nor were there any obvious changes in cells that had been pre-permeabilized with saponin to wash out the cytosolic pool of adaptors and enhance the visualization of membrane labeling (Figure 3A). We therefore used quantitative electron microscopy (EM) to investigate clathrin coat formation at the cell surface. To distinguish plasma membrane-associated CCPs from free CCVs, cells were fixed in the presence of ruthenium red. This heavy metal stain is absorbed onto the plasma membrane where it forms an electron-dense layer visible by EM but is excluded from free CCVs that have already pinched off. Therefore, any clathrin-coated and ruthenium red-positive structures at or near the plasma membrane are defined as CCPs (Figure 3B). In cells depleted of auxilin 1 and auxilin 2, the number of shallow and curved CCPs was reduced to 45% and the number of deeply invaginated CCPs to 66%, relative to control cells. Although reduced in number, the pits appear to have normal morphology. In cells depleted of dynamin, the number of shallow and curved CCPs was reduced to 25% of control levels; however, we observed a fourfold increase in the number of deeply invaginated CCPs (Figure 3B,C). This agrees with previous studies of a dominant-negative dynamin mutant and with the proposed role of dynamin in the fission of CCPs (23).

In yeast, knockout of auxilin causes the accumulation of free CCVs (16). However, we found that in auxilin 1/auxilin 2-depleted HeLa cells, there was actually a decrease in the number of free plasma membrane-derived CCVs defined as ruthenium red-negative, clathrin-coated structures within 200 nm of the cell surface (52%, relative to control cells). As expected, there was also a reduction in the number of free CCVs in dynamin-depleted cells (36% of control levels).

### Auxilin depletion inhibits intracellular trafficking

**Figure 4:**
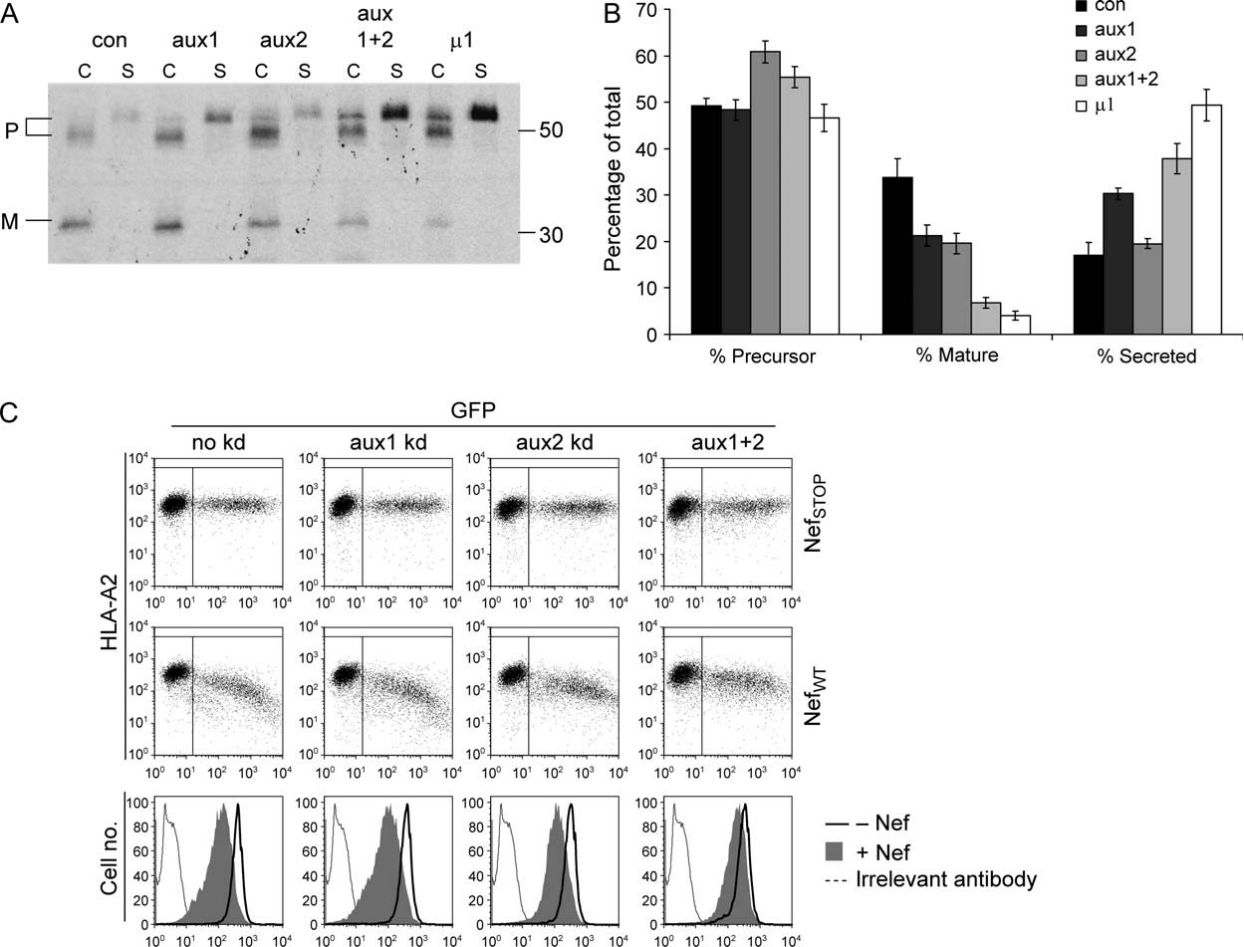
Auxilin depletion affects clathrin-mediated intracellular sorting A) Control cells, cells depleted of both auxilins and cells depleted of AP-1 μ1A were pulse-chased with ^35^S, and both cell-associated (C) and secreted (S) cathepsin D was immunoprecipitated. The positions of the precursor (P) and mature (M) forms of the enzyme are marked. B) Results from three separate experiments were pooled; error bars show the standard error of the mean. The ratio of secreted cathepsin D precursor to total cathepsin D is 0.17 in control, 0.35 in auxilin double-depleted and 0.51 in the AP-1 μ1A-depleted cells. C) HeLa cells stably expressing HLA-A2 were depleted of auxilin 1, auxilin 2 or both by siRNA and then transfected with either a control plasmid-encoding GFP alone (Nef_STOP_) or a plasmid-encoding wild-type Nef as a bicistronic message with GFP (Nef_WT_). Flow cytometry was used to assay for MHCI downregulation using anti-HLA-A2 bound at 4°C. Representative plots show dose-dependent downregulation of MHCI in control cells, which is reduced in auxilin-depleted cells.

Depletion of auxilin 2 has been reported to affect lysosomal sorting by altering the localization of mannose 6-phosphate receptors (18) and delaying the maturation of cathepsin D (7,21). To assess the effects of knocking down both auxilins, we performed a cathepsin D sorting assay. HeLa cells were pulse-chased with ^35^S, and cathepsin D was immunoprecipitated both from the medium and from the cells. Figure 4A shows a representative autoradiograph, and Figure 4B shows the quantification of three such experiments. Depletion of either auxilin 1 or auxilin 2 has only a small effect on cathepsin D sorting. However, depletion of both auxilins results in substantial missorting of cathepsin D, as evidenced both by the reduction in mature enzyme and by the increase in secretion of cathepsin D precursor. A similar phenotype is seen in AP-1 depleted cells (Figure 4B).

Another AP-1-dependent pathway is the downregulation of major histocompatibility complex class I (MHCI) from the cell surface by Nef, a protein encoded by HIV. Knockdown of either AP-1 or clathrin inhibits the Nef-induced rerouting of MHCI to endosomes and lysosomes (24,25), whereas knockdown of AP-2 enhances the effect of Nef on MHCI (25). To investigate the role of auxilins in this pathway, we used a fluorescence-activated cell sorter (FACS)-based assay. Control (i.e. no knockdown) and siRNA-treated cells were transiently transfected with plasmids encoding either green fluorescent protein (GFP) alone or GFP and Nef as a bicistronic message, and the cells were scored for GFP expression and surface MHCI. Depletion of either auxilin 1 or auxilin 2 had only a slight effect, but the simultaneous depletion of both auxilins had a stronger inhibitory effect on Nef activity (Figure 4C).

Together, these results demonstrate that the two auxilins have overlapping functions not only at the plasma membrane but also in intracellular trafficking events and that in HeLa cells depletion of both auxilins is required to impair both the sorting of lysosomal hydrolases and Nef-induced downregulation of MHCI.

### Effects of auxilin depletion on the localization of clathrin and adaptors on intracellular membranes

**Figure 5:**
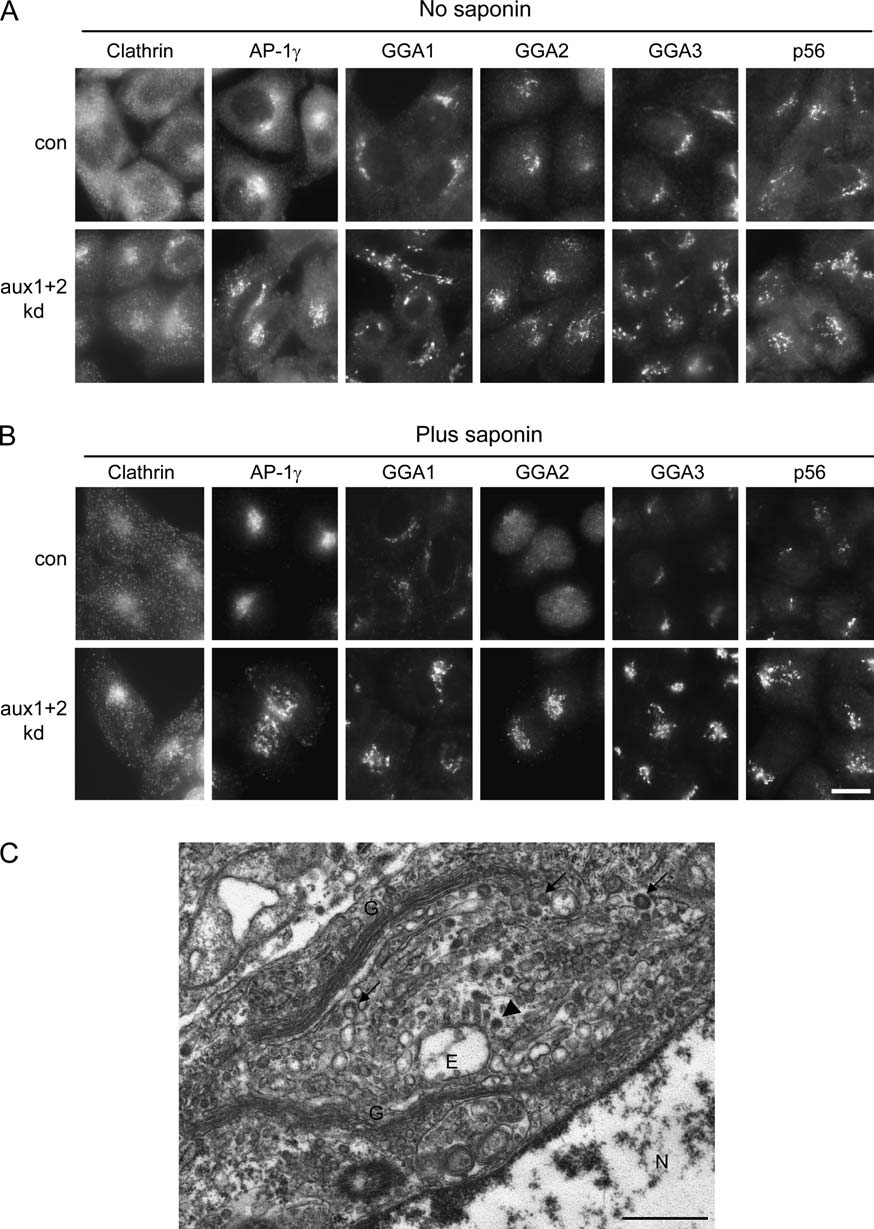
Effects of auxilin depletion on clathrin and adaptors associated with intracellular membranes Control cells or cells depleted of both auxilins were incubated without (A) or with (B) 0.05% saponin prior to fixation and then labeled with antibodies against clathrin, AP-1γ, GGA1, GGA2, GGA3 and the GGA-binding partner p56. Scale bar: 20 μm. C) Representative electron micrograph showing the Golgi region in a HeLa cell depleted of auxilin 1 and auxilin 2. Cells were incubated with 0.05% saponin prior to fixation. Note that the morphology of the Golgi appears normal and that there are a number of clathrin-coated budding profiles associated with the TGN (arrows) and with tubules emanating from endosomes (arrowhead). E, endosome; G, Golgi; N, nucleus. Scale bar: 500 nm.

Different effects of auxilin 2 knockdown have been reported on the localization of clathrin and adaptor proteins associated with intracellular membranes. Lee et al. (18) reported the loss of AP-1 and Golgi-localized γ-ear containing ARF-binding proteins (GGA) immunostaining, while Kametaka et al. (7) reported no effect on AP-1. We used immunofluorescence to investigate the effects of an auxilin double knockdown on the localization and labeling intensity of clathrin, AP-1, GGAs and p56 (the best characterized GGA-binding partner) (Figure 5). We observed relative increases in the labeling intensities of clathrin, AP-1, GGA1, GGA2, GGA3 and p56 associated with perinuclear membranes (Figure 5A). Using saponin pre-permeabilization to wash out the cytosolic pool of adaptors, we observed even more pronounced increases in the labeling intensities of GGAs and their binding partner p56 (Figure 5B). We have previously reported that freeze thawing causes a rapid loss of GGA1 from the perinuclear region of the cell because of the lability of their association with membranes (26). Treatment with saponin prior to fixation caused a similar loss of GGA and p56 immunofluorescence staining (compare Figure 5A with Figure 5B). In contrast, in auxilin double-depleted cells, the GGAs and p56 were retained on the membrane even after saponin pre-permeabilization. These results suggest that in the absence of auxilin 1 and auxilin 2, the GGAs (and consequently their binding partner p56) associate more stably with membranes. The association was still sensitive to treatment with the drug Brefeldin A and therefore dependent on ARF1 (ADP ribosylation factor 1) (Figure S2). Using EM to investigate the morphology of the Golgi region at the ultrastructural level, we observed that the Golgi stack appears normal and that there are numerous clathrin-coated budding profiles associated with the *trans* Golgi network (TGN) and endosomes (Figure 5C).

**Figure 6:**
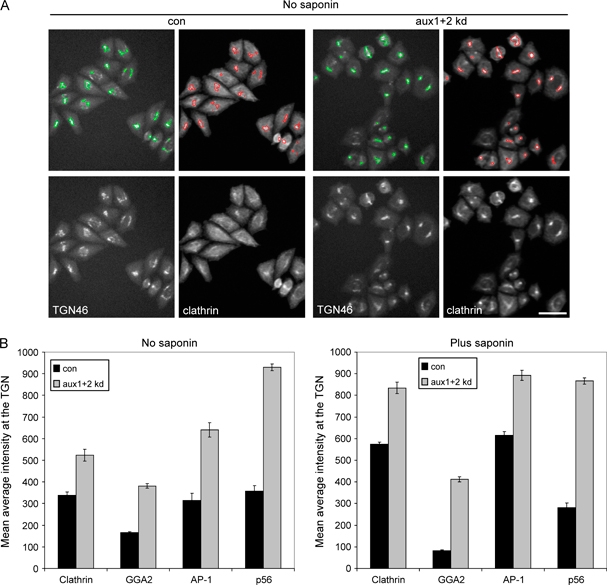
Quantification of effects of auxilin knockdown on intracellular coat proteins Control cells or cells depleted of auxilin 1 and auxilin 2 were incubated with or without 0.05% saponin prior to fixation, double labeled with antibodies against TGN46 and either clathrin, AP1-γ, GGA2 or the GGA-binding partner p56 and imaged using an automated microscope. A) Representative images of clathrin labeling in the absence of saponin. The top panels show the automated masking of the TGN. Scale bar: 50 μm. B) For each condition, data from more than 2500 cells (total) from 10-well repeats were pooled, the mean average intensity was determined and data were corrected for changes in TGN46 labeling. Error bars show the standard error of the mean. All the coat proteins show increased fluorescence in the TGN region after auxilin knockdown.

The increases in labeling of clathrin, AP-1, GGAs and p56 on intracellular membranes in auxilin-depleted cells were quantified using an automated microscope, which enabled us to sample more than 2500 randomly chosen cells for each condition. Clathrin labeling is shown as an example of how the data were collected (Figure 6A). We found increases in labeling intensity of 1.20-fold for clathrin, 1.58-fold for AP-1, 1.71-fold for GGA2 and 2.02-fold for p56. Using cells pre-permeabilized with saponin, the increase became even more apparent for GGA2 (3.90-fold) and for p56 (2.40-fold) (Figure 6B).

Together, these results show that auxilin depletion increases the relative association of clathrin, AP-1, GGAs and p56 with intracellular membranes. As Hsc70 and auxilin are implicated in the ATP-dependent rapid exchange of free clathrin in the cytosol with clathrin in pits and buds (19), it is likely that the stabilization is because of alterations in the cycling of coat proteins on and off membranes.

### Dynamics of clathrin exchange

**Figure 7:**
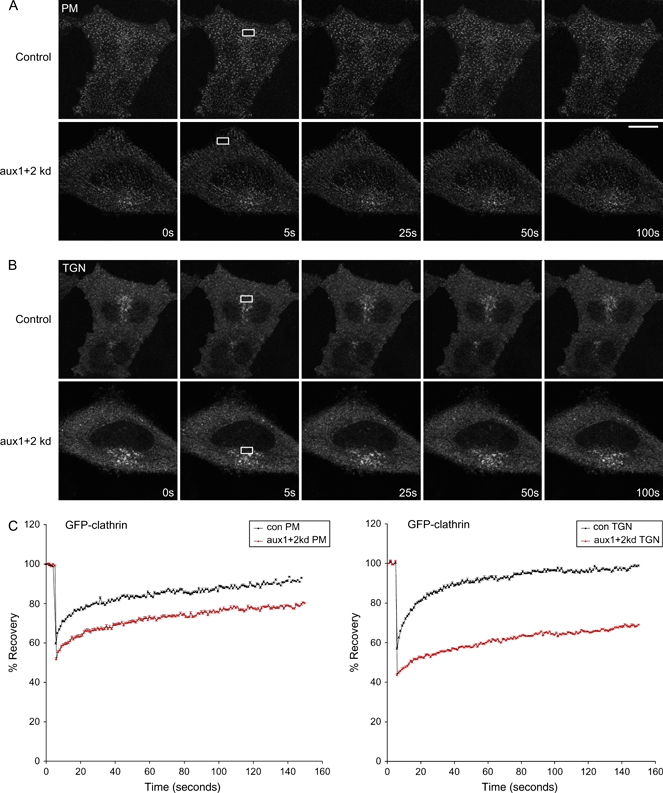
Auxilin depletion changes clathrin dynamics Control cells or cells depleted of both auxilins were transfected with GFP-tagged clathrin light chain. The plasma membrane (A) or a perinuclear region (B) was photobleached and fluorescence allowed to recover for 150 seconds. Scale bar: 10 μm. C) Fluorescence recovery was adjusted for background and bleaching and then normalized to percentage recovery. Fourteen data sets were pooled and the mean results were plotted; error bars show the standard error of the mean.

To assess the dynamics of clathrin exchange, cells were transfected with GFP-clathrin light chain and analyzed by fluorescence recovery after photobleaching (FRAP) at the plasma membrane (Figure 7A) and in the perinuclear region (Figure 7B). Cells depleted of both auxilin 1 and auxilin 2 showed a reduced fluorescence recovery after 150 seconds compared with control cells at both locations. In the auxilin-depleted cells, there was 80.2 ± 0.4% recovery at the plasma membrane and 69.1 ± 0.3% recovery in the perinuclear region, while in control cells, there was 93.1 ± 0.3% recovery at the plasma membrane and 99.0 ± 0.3% recovery in the perinuclear region (Figure 7C), which is consistent with the presence of an immobilized pool of clathrin on the membrane in the auxilin-depleted cells. In addition, the fluorescence recovery was slower in auxilin-depleted cells, with a half-time (*t*_1/2_) of 29.1 ± 1.4 seconds at the plasma membrane compared with a *t*_1/2_ of 24.4 ± 2.7 seconds in control cells, and a *t*_1/2_ of 40.6 ± 2.4 seconds in the perinuclear region compared with a *t*_1/2_ of 14.4 ± 1.1 seconds in control cells. Together, these results show that auxilin depletion causes changes in clathrin dynamics.

### CCV fractions from auxilin-depleted cells

**Figure 8:**
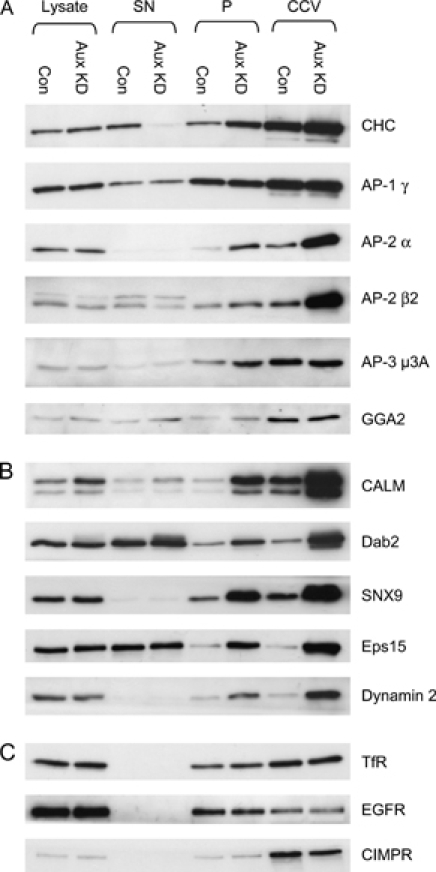
CCV fractions from auxilin-depleted cells have increased clathrin and AP-2 content Post-nuclear supernatants of whole cell lysates from control (con) and auxilin double-depleted (aux KD) HeLa cells were centrifuged at 135 000 × ***g*** to yield a soluble fraction (SN) and a high-speed pellet (P). A CCV-enriched fraction (CCVs) was prepared from the pellet by two further rounds of differential centrifugation. Samples were analyzed by western blotting. The following protein quantities were loaded per lane: Lysate, 10 μg; SN, 10 μg; P, 5 μg; CCV, 2.5 μg. A) Clathrin and adaptor proteins. B) Endocytic accessory proteins. C) Cargo proteins. Note that the amount of protein in the CCV fractions represents only a small proportion of the protein present in the SN and P fractions (<1%); hence, large changes of abundance in the CCV fraction are not necessarily paralleled by equally large changes in the SN and P fractions.

Knockout of auxilin in yeast causes an accumulation of free CCVs and an increase in the yield of CCVs from cell homogenates (16). In HeLa cells, auxilin 2 knockdown causes overexpressed clathrin–GFP to aggregate in the cytosol, but no accumulation of free CCVs has been reported (18). To test how an auxilin 1/auxilin 2 double knockdown affects the yield of CCVs from HeLa cells, we prepared CCV-enriched fractions from control and auxilin-depleted cells, using our previously established fractionation protocol (22,27). Samples were adjusted to equal protein concentrations and analyzed by western blotting (Figure 8). CCV fractions from auxilin 1/auxilin 2-depleted cells showed a significant increase in clathrin relative to control CCV fractions (∼1.5-fold; Figure 8A). While levels of adaptor proteins AP-1, AP-3 and GGAs were unaffected, there was a large and selective increase in AP-2 (more than fivefold; Figure 8A). This increase was mirrored by AP-2-associated proteins, including CALM, Dab2, SNX9, Eps15 and dynamin 2 (Figure 8B). Intriguingly, there was no concomitant increase in typical endocytic cargo molecules, such as EGF and transferrin receptors (Figure 8C). We even observed a small but consistent decrease in CIMPR, a cargo molecule of both endocytic and intracellular CCVs (Figure 8C). In addition, auxilin double knockdown caused a strong reduction in the amount of clathrin in our high-speed supernatant (Figure 8A), indicating that there is much less free clathrin in the cytosol.

**Figure 9:**
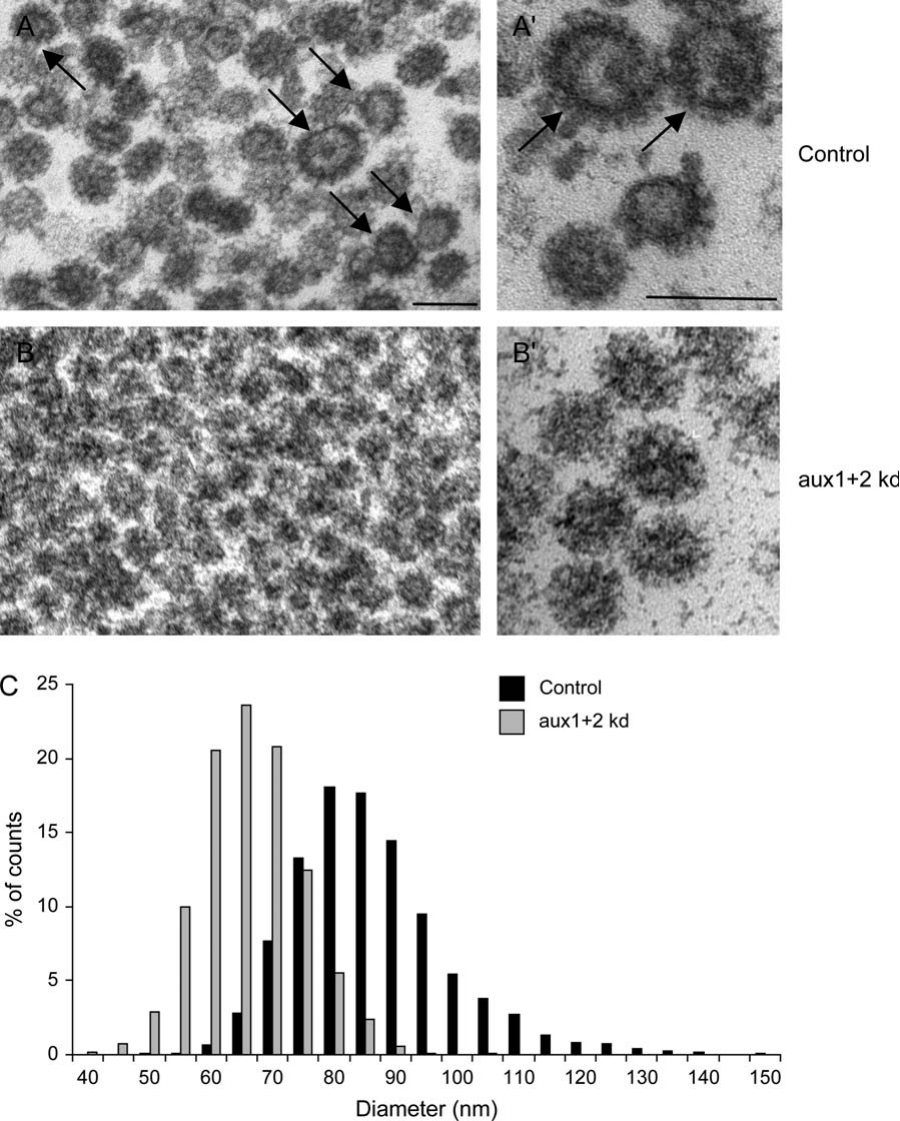
EM analysis of clathrin-coated structures isolated from auxilin-depleted cells CCV fractions were prepared from control and auxilin 1/auxilin 2-depleted cells. The final pellets were fixed and embedded, and ∼70 nm sections were cut perpendicular to the pellet. Electron micrographs show clathrin-coated structures isolated from control cells (A and A′) and auxilin-depleted cells (B and B′). Clathrin-coated structures from control cells (A) appear more heterogeneous in size than clathrin-coated structures from auxilin-depleted cells. A membrane is frequently observed in control CCVs [arrows in (A) and (A′)], whereas clathrin-coated structures from auxilin-depleted cells mostly seem to lack membranes (B′). Scale bars: 100 nm. C) Size distribution of clathrin-coated structures isolated from control and auxilin-depleted cells. In total, the mean diameters of 3369 and 2534 structures were measured for control cells and auxilin-depleted cells, respectively. The size distribution is expressed as a percentage of the total number measured for each condition, with 5 nm binning. The average diameter of control CCVs is 84 nm (SD = 13 nm), whereas the average diameter of clathrin-coated structures in auxilin-depleted cells is 64 nm (SD = 9 nm).

The selective increase in clathrin, AP-2 and associated proteins in the CCV fraction suggests that the auxilin double knockdown causes a stabilization and subsequent accumulation of endocytic CCVs. However, the lack of increase in cargo molecules indicates that these may be CCVs that have budded without concentrating cargo. Alternatively, auxilin double knockdown may cause the formation of membraneless clathrin–adaptor cages that have similar fractionation properties as CCVs. To distinguish between these possibilities, we analyzed our CCV fractions by EM. In preparations from control cells, CCVs were readily identified as spherical or ellipsoid structures of variable size with the bristle border that is characteristic of the clathrin coat in thin sections (Figure 9A). A membrane was clearly visible inside many of the CCVs (Figure 9A, arrows). In contrast, the clathrin-coated structures isolated from auxilin double-depleted cells appear to be smaller, more electron dense and more uniform in size; in addition, a membrane was rarely visible in these structures (Figure 9B). Quantitative analysis showed that auxilin 1/auxilin 2 depletion causes a shift in the size distribution of coated structures (Figure 9C). The control CCV population has a mean diameter of 84 nm, with a standard deviation (SD) of 13 nm (*n* = 3369). The clathrin-coated structures from auxilin-depleted cells are significantly smaller with a mean diameter of 64 nm and are also more homogeneous in size, with a SD of 9 nm (*n* = 2534). Similar size distributions were observed when the CCV fractions were analyzed by negative staining (unpublished data). The clathrin-coated structures from auxilin-depleted cells also tend to be more uniformly round, whereas in the control preparations, there is a higher frequency of irregularly shaped, incompletely coated vesicles, which may represent sheared-off clathrin-coated buds (Figure 9A and Figure S3). Together, these data suggest that the auxilin double knockdown causes the accumulation of small, homogeneous, clathrin-coated structures that resemble CCVs in morphology but lack membranes.

**Figure 10:**
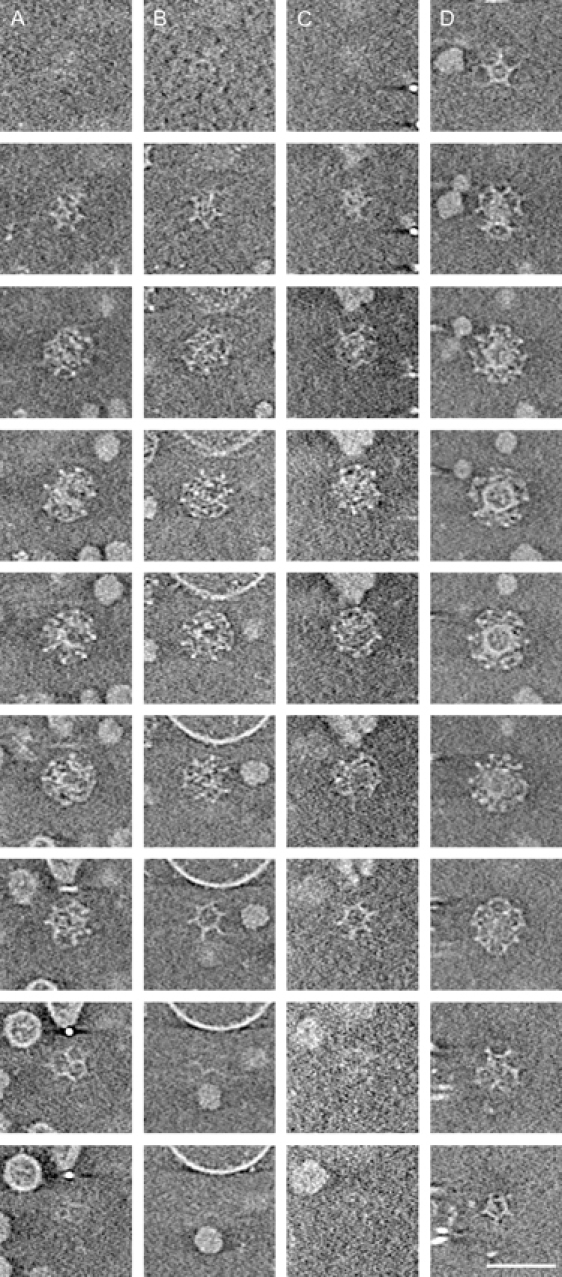
Tomography of vitrified clathrin-coated structures isolated from auxilin 1/auxilin 2-depleted HeLa cells Panels A–C show tomographic slices of membraneless clathrin-coated structures. Panel D shows a tomograph of a membrane-containing CCV for comparison. The thickness of each slice is 14.3 Å; images correspond to every eighth slice of the tomographic stack. Scale bar: 100 nm.

To confirm that the clathrin-coated structures from auxilin-depleted cells are indeed membraneless, we used cryo-electron tomography to obtain three-dimensional (3D) reconstructions of individual frozen-hydrated particles. Images in a tilt series (recorded as described in *Materials and Methods*) were aligned with each other to calculate the electron tomograms. We found that the majority of the reconstructed clathrin-coated structures are devoid of a membrane vesicle (Figure 10A–C). Comparison with tomographic slices of isolated CCVs containing a membrane vesicle (Figure 10D) also shows that most of the membraneless clathrin-coated structures are smaller than CCVs, consistent with the thin section data, and that they contain a dense core.

### Membraneless clathrin coats are present in intact auxilin-depleted cells

**Figure 11:**
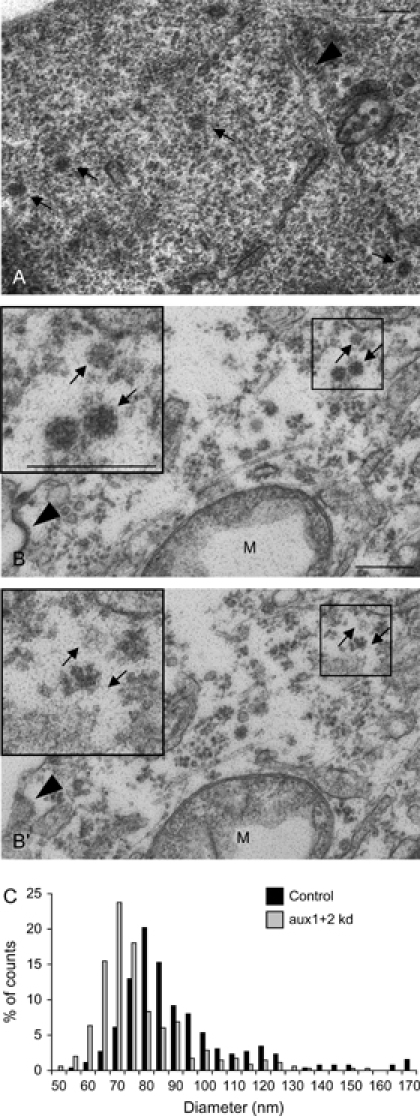
EM analysis of intracellular clathrin-coated structures in auxilin-depleted cells Cells depleted of auxilin 1 and auxilin 2 were treated either without (A) or with (B) 0.05% saponin, then fixed either with (A) or without (B) including 1% tannic acid in the final step and embedded in resin. Sections of ∼70 nm were cut, and for B and B′, two consecutive serial sections are shown. Note the presence of small electron-dense clathrin-coated profiles in the cytoplasm that appear to be devoid of membrane. The arrowhead in (A) shows a 25 nm microtubule for size comparison; the arrowhead in (B/B′) shows a CCP. Three membraneless electron-dense clathrin-coated profiles are enlarged in the insets. Unlike the CCP, they are only visible in a single section. Scale bars: 100 nm (A); 250 nm (B); M, mitochondrion. C) Size distribution of clathrin-coated structures in the cytoplasm of control and auxilin-depleted cells. The diameter of 258 and 344 clathrin-coated structures was measured for control cells and auxilin-depleted cells, respectively. The size distribution is expressed as a percentage of the total number measured for each condition. The average diameter of clathrin-coated structures was 74 nm (SD = 15 nm) in auxilin-depleted cells and 88 nm (SD = 21 nm) in control cells.

The membraneless structures observed in the auxilin double knockdown are reminiscent of clathrin coats assembled *in vitro*(28), suggesting that they might have formed during the fractionation procedure. To test whether these structures are also present in intact HeLa cells, we analyzed the cells by EM. Small, electron-dense and seemingly membraneless clathrin-coated structures were frequently observed in the cytoplasm of auxilin-depleted cells (Figure 11A). For a quantitative analysis, control and auxilin-depleted HeLa cells were pre-permeabilized with saponin to reduce cytosolic background (Figure 11B,C). Again, we observed a shift in size distribution: clathrin-coated structures from auxilin-depleted cells have a mean diameter of 74 ± 15 nm versus 88 ± 21 nm from control cells [*n* = 344 and 258, respectively (Figure 11C)]. Both mean diameters are larger than those that we measured when we analyzed our CCV fractions, probably because many of the structures in intact cells are clathrin-coated buds, not free CCVs. Nevertheless, the mean diameter of clathrin-coated structures is significantly smaller in the auxilin-depleted cells than in the control cells. In addition, in control cells, the majority (67%) of the clathrin-coated structures have clearly visible membranes. In contrast, in auxilin-depleted cells, only 24% of clathrin-coated structures have visible membranes.

Although clathrin-coated structures without detectable membranes may represent genuine empty clathrin cages, it is also possible that they are a result of grazing sections of CCVs or clathrin-coated buds. Indeed, the latter possibility may explain why even in control cells a proportion of clathrin-coated structures have no visible membrane. To determine whether the seemingly membraneless structures in auxilin-depleted cells actually have membranes outside of the plane of section, we analyzed serial sections. We found that the majority of the clathrin-coated structures in auxilin-depleted cells were contained within a single 70-nm thick section (Figure 11B,B′, see arrows and inset), demonstrating that they are smaller than a CCP or bud, which can usually be followed through two or more serial sections (Figure 11B,B′, see arrowhead). This suggests that most of the membraneless structures observed in auxilin-depleted cells are indeed free clathrin cages.

## Discussion

Previous attempts to study the role of auxilin in mammalian cells have produced somewhat conflicting results that did not correlate with the phenotypes of auxilin inactivation in other organisms. Most of the mammalian studies were based on RNAi depletion of auxilin 2 in HeLa cells, which caused only mild defects in endocytosis and intracellular sorting, inconsistent effects on clathrin dynamics and perhaps most disappointingly no accumulation of CCVs (7,18,21,29). One underlying problem with the majority of these studies has been the assumption that HeLa cells express only the ubiquitous isoform of auxilin, auxilin 2. In this study, we show that HeLa cells express not only the ubiquitous auxilin 2 but also the ‘neuron-specific’ isoform, auxilin 1. This has allowed us to uncover further roles for auxilin, in addition to its well-defined role in the uncoating of CCVs, by using RNAi to deplete both isoforms.

### Auxilin 1 and auxilin 2 functionally complement each other

Knockdown of auxilin 2 alone has a weak inhibitory effect on endocytosis (18,21). In the present study, we demonstrate that both auxilin 1 and auxilin 2 contribute to clathrin-mediated endocytosis because only the combined depletion of both auxilins inhibits the uptake of transferrin and EGF. We also show that both auxilin 1 and auxilin 2 contribute to the correct sorting of cathepsin D and to Nef-mediated downregulation of MHCI. In agreement with these observations, our data suggest that auxilin 1 and auxilin 2 are present in similar quantities in HeLa CCVs. Thus, auxilin 1 and auxilin 2 have overlapping functions and can substitute for each other in sorting events both at the plasma membrane and on intracellular membranes.

### Auxilin depletion has different effects on clathrin association with intracellular membranes and the plasma membrane

Auxilin has been proposed to function at various stages during the CCV cycle, including clathrin recruitment (18), clathrin remodeling (19), vesicle scission (20) and vesicle uncoating. Despite the disruption in both endocytic and TGN/endosome clathrin-mediated trafficking in cells depleted of both auxilins, the knockdown appears to have opposite effects on the association of clathrin with the plasma membrane and with intracellular membranes. In auxilin-depleted cells, there was a ∼50% drop in the number of clathrin-coated profiles at the plasma membrane, whereas there was a significant increase in clathrin and AP-1/GGA labeling on intracellular membranes. We can only speculate as to why these differences exist, but the two most likely explanations are differences in how the auxilins act on AP-1 and AP-2 or mechanistic differences in adaptor protein recruitment. For example, auxilin 2 has a serine/threonine kinase domain that is not found in auxilin 1, which is homologous to the kinase domain of adaptor-associated kinase 1 (AAK1) (30). Both auxilin 2 and AAK1 can phosphorylate the μ2 subunit of AP-2, so it is possible that the kinase activities of AAK1 and auxilin 2 can functionally substitute for each other at the plasma membrane but not on intracellular membranes. Alternatively (or in addition), the different mechanisms employed by AP-2 and AP-1/GGAs to bind to their target membranes, involving different phosphoinositides and ARF guanosine triphosphatases, may influence the way they respond to auxilin depletion.

### What is the function of auxilin in the CCV cycle?

If the sole function of auxilin is to act as a cofactor for Hsc70 in the uncoating of CCVs, then it might be expected that knocking down auxilin would lead to an accumulation of CCVs. Indeed, this phenotype has been reported in auxilin-depleted yeast (16). However, by EM, we did not observe an accumulation of free endocytic CCVs, defined as ruthenium red-negative clathrin-coated structures within 200 nm of the plasma membrane. In contrast, we did find that CCV fractions from auxilin-depleted cells were significantly enriched in clathrin and components of the endocytic machinery, relative to CCV fractions from control cells. At first, these observations seemed to contradict each other, but several observations have led us to conclude that the clathrin-coated structures that accumulate in auxilin-depleted cells are assembled clathrin cages that are devoid of membrane. Firstly, while there is an increase in clathrin and AP-2 in CCV fractions from auxilin-depleted cells, cargo proteins such as the transferrin receptor, EGF receptor or CIMPR are not increased. Secondly, in auxilin-depleted cells, clathrin redistributes from ∼50% cytosolic to being almost completely pelletable. Thirdly, EM studies show that the mean size of purified clathrin-coated structures from auxilin-depleted cells is substantially smaller than that from control cells and similar in size to cages assembled *in vitro* from clathrin and AP complexes (28). Most importantly, the majority of clathrin-coated structures isolated from auxilin-depleted cells appear to be devoid of membrane.

The increase in the amount of AP-2 in CCV fractions from auxilin-depleted cells correlates with the observed decrease in plasma membrane-associated CCPs. In contrast, AP-1 is not lost from intracellular membranes and does not accumulate in CCV fractions. Hence, biochemical and imaging data are consistent, all indicating that membraneless clathrin cages form in auxilin-depleted HeLa cells. This in turn suggests that in addition to its role in uncoating, a major function of auxilin is to prevent the nonproductive assembly of clathrin, as has been suggested from *in vitro* studies (31).

Could the formation of cages lead to a sequestration of endocytic machinery and thus cause the observed block in transferrin and EGF uptake? Probably not, given that we observed only a 50% drop in the number of CCPs. Hence, it seems more likely that the endocytic and intracellular blocks are a consequence of altered clathrin dynamics.

### Evidence for *in vivo* self-assembly of clathrin into membraneless coats

At concentrations above ∼50 μg/mL, clathrin has the propensity to self-assemble *in vitro* into structures of 70–125 nm, termed ‘cages’ or ‘baskets’ (28). This assembly is greatly facilitated by the presence of adaptor proteins, which also cause the cages to become smaller and more uniform in size (28). Although the formation of membraneless clathrin cages *in vitro* is a well-documented phenomenon, to our knowledge, free cages have never before been observed *in vivo*. Under certain nonphysiological conditions (potassium depletion, growth in hypertonic medium and acidification of the cytoplasm), microcages have been seen to form around CCPs (32,33), but the structures that we observe in auxilin-depleted cells do not accumulate at the plasma membrane but are present throughout the cytoplasm. It has been suggested that the concentration of free cytosolic clathrin is too low to allow spontaneous self-assembly in cells (34). However, using enzyme-linked immunosorbent assays to measure clathrin concentration as a percentage of total cell protein in different cells and tissues, Goud et al. (35) have arrived at estimates of 0.18 ± 0.02% (of which 59% is unassembled) and 0.16 ± 0.07% (of which 45% is unassembled) in Vero cells and X63Ag8 cells, respectively (HeLa cells were not included in the study). If the total protein concentration of a cell is ∼180 mg/mL (36), the cytosolic clathrin concentration in a typical tissue culture cell would be ∼100–200 μg/mL, which is certainly high enough for spontaneous self-assembly to occur if there were no inhibitory mechanisms in place.

Interestingly, Newmyer and Schmid (37) have observed that in HeLa cells overexpressing dominant-negative Hsc70, clathrin shifts from the unassembled pool to the assembled pool, as determined by subcellular fractionation, without a concomitant shift of CCV cargo molecules. This led them to speculate that in the absence of Hsc70’s chaperoning activity, clathrin may form empty cages. However, the proposed cages were neither visualized nor isolated. In this study, we used EM to present the first direct evidence for *in vivo* assembly of clathrin into cages that are distinct from CCVs. In auxilin-depleted HeLa cells, small and membraneless clathrin-coated structures accumulate in the cytoplasm. These structures are smaller than CCVs from control cells and show a narrow size distribution similar to that observed for cages assembled *in vitro*. Their electron-dense appearance, in particular by EM tomography, suggests that they are packed with proteins. This is further supported by the biochemical fractionation data that show a greatly increased AP-2-to-clathrin ratio in CCV fractions from auxilin-depleted cells. Collectively, these observations suggest that the clathrin cages incorporate AP-2 and associated proteins at higher than normal stoichiometry. Possibly, the absence of a membrane and of cargo proteins puts fewer spatial constraints on cage formation and leads to a higher packing density of clathrin-binding proteins.

Intriguingly, it appears that AP-2 and endocytic accessory factors are preferentially packaged into the membraneless cages, relative to AP-1 and AP-1-binding proteins. We propose that at least two factors may be responsible for this striking difference. Firstly, there are many more known binding partners for AP-2 than for AP-1 (38). Secondly, the numerous AP-2-associated proteins form strong protein–protein interaction networks through SH3/proline-rich domains as well as EH domains/NPF motifs, whereas this is not the case for the known AP-1-associated proteins (38). Hence, although both AP-1 and AP-2 and their binding proteins may initially be recruited to the membraneless clathrin cages, the stronger and more extensive interaction network of AP-2 will outcompete the AP-1 network, resulting in a skewed AP-2 to AP-1 ratio.

### Conclusion and outlook

In this study, we have shown that HeLa cells express both isoforms of auxilin and that these are functionally complementary in both endocytosis and intracellular clathrin-mediated trafficking. In view of these findings, the existing discrepancies between the mammalian and nonmammalian auxilin literature are likely to be resolved. Furthermore, we have demonstrated for the first time that in the absence of auxilin, clathrin and adaptor proteins assemble into membraneless cages in intact HeLa cells, shedding light both on a novel role for auxilin and on clathrin assembly dynamics *in vivo*. Finally, auxilin depletion causes the accumulation of clathrin cages that are selectively packed with endocytic adaptors and accessory factors. Hence, auxilin knockdown provides a convenient tool for specifically increasing the yield of AP-2-associated proteins during biochemical fractionation and will facilitate future proteomic investigations of the endocytic clathrin machinery.

## Materials and Methods

### Antibodies and blotting

Rabbit polyclonal antibodies against auxilin 1 (which also recognizes auxilin 2) (9) and GGA3 (39) were kind gifts from Sanja Sever (Harvard Medical School) and Juan Bonifacino (National Institutes of Health), respectively, and those against GGA1, GGA2, p56, SNX9, clathrin, AP-2α, AP-2β2, AP-3μ3 and CIMPR were raised in house and have already been described (22,40–42). Mouse monoclonal antibodies against clathrin (X22) and AP-2α (AP-6) were kind gifts from Frances Brodsky (University of California, San Francisco). Commercial antibodies against Dab2, MHCI (BB7.2), TGN46, AP-1γ (Mab100.3), CALM, auxilin 2, dynamin 2, CD8, transferrin receptor and EGF receptor were purchased from Santa Cruz, BD Biosciences, Serotec, Sigma-Aldrich, Santa Cruz, MBL Co Ltd, Abcam, Ancell, Zymed and Santa Cruz, respectively. SDS–PAGE and western blots were performed using standard methods. The bands were detected using enhanced chemiluminescence and quantified using Image J software (http://rsb.info.nih.gov/ij). The human tissue and cell line blots (INSTA-Blot) were purchased from Calbiochem.

### Immunolocalization and ligand uptake

For immunofluorescence, HeLa M cells (43) were fixed with 3% paraformaldehyde (PFA), followed by permeabilization with 0.1% Triton-X-100. For some experiments, cells were pre-permeabilized with a 30-second wash with 0.05% saponin in cytosol buffer (25 mm HEPES-KOH, pH 7.4, 25 mm KCl, 2.5 mm magnesium acetate, 5 mm EGTA and 150 mm K-glutamate) prior to fixation (44,45). Primary antibodies are described above; secondary antibodies were purchased from Molecular Probes. For ligand uptake experiments, cells were pre-incubated at 37°C for 30 min in serum-free medium containing 0.5% BSA and 25 mm HEPES. Cells were then incubated at 4°C with 10 μg/mL Alexa Fluor 594-labeled human transferrin or 400 ng/mL Alexa Fluor 488-labeled EGF (Molecular Probes) for 30 min. Excess label was washed off and the cells were warmed to 37°C for 10 min to allow uptake of the ligand and then fixed. The cells were imaged with a Zeiss Axiovert 200 inverted microscope using a Zeiss Plan Achromat ×63 oil immersion objective, 1.40 numerical aperture, a Hamamatsu ORCA-ER2 camera and improvision openlab software.

### Quantification of fluorescence

Quantification of fluorescence intensity of various proteins at the TGN was measured using an automated ArrayScan VTI microscope (Cellomics/Thermo-Fisher) and the TargetActivation.V2 assay algorithm. Cells plated onto 96-well Nunclon Surface plates were co-stained using 4′-6-diamidino-2-phenylindole (DAPI), a sheep polyclonal antibody against TGN46, and rabbit polyclonal antibodies against a variety of proteins of interest, followed by Alexa Fluor 488-labeled donkey anti-sheep immunoglobulin G (IgG) and Alexa Fluor 594-labeled donkey anti-rabbit IgG. The camera was automated to identify cells using the DAPI channel, to mask a region of interest using TGN46 labeling (object identification using the Isodata Threshold method), to correct for background locally within each field and then to measure the average fluorescence intensity in the Alexa 594 channel. For each condition, 5000 objects of interest were quantified spread over 10-well repeats, which equates to at least 2500 cells (where not all TGNs were defined by a single mask). The cells were imaged with a modified Zeiss Axiovert 200M inverted microscope, a Zeiss ×20/0.45 Achroplan objective, a Hamamatsu ORCA-ER camera and arrayscan software. Because there was an increase of 1.29 in the mean size/labeling intensity of TGN46 in the auxilin-depleted cells, data collected from the other channels were corrected for the difference in TGN46 labeling by division by 1.29.

### Fluorescence recovery after photobleaching

Cells were transfected with GFP-clathrin light chain using FuGENE 6 (Roche) for 48 h. A defined region was photobleached, resulting in a 40–50% reduction in the pre-bleach fluorescence intensity. The cells were imaged with a Zeiss LSM510 META confocal microscope using a Zeiss ×63/1.4 Plan-Apochromat oil immersion objective and lsm software (version 4.0). The data were corrected for bleaching by dividing the intensity of the bleached area by that of an unbleached area and then normalized. Each curve was fitted to the equation *Y* = *Y*_max_ (1−exp(−*KX*) using graphpad prism software to determine the *t*_1/2_ of recovery. For each experimental condition, 14 data sets were averaged.

### Internalization and intracellular sorting assays

Ligand uptake assays were performed as described by Motley et al. (46). Cathepsin D sorting was assayed essentially as described by Davidson (47), using 5 μL ^35^S Promix (Amersham Biosciences) per milliliter of medium. The cells were pulse labeled for 15 min and chased for 2.5 h in the presence of 5 mm mannose 6-phosphate before immunoprecipitation using rabbit anti-human cathepsin D (DAKO). Quantifications were carried out using a Packard Cyclone phosphorimager. A FACS-based assay was used to assess Nef-mediated MHCI downregulation, as previously described (25).

### CCV isolation and RNAi

The isolation of CCVs from control and auxilin-depleted HeLa cells was performed as previously described (22,27). Auxilin 1 was depleted using oligo-2 (UAUGUUACCUCCAGAAUUA; D-009885-02) from the siGENOME SMARTpool (Dharmacon), which was found to be the least toxic of the four to the cells (although the phenotype was found to be reproducible with oligo-3). Dynamin 2 and auxilin 2 were depleted using ON-TARGETplus SMARTpool (L-004007-00) and siGENOME SMARTpool (M-005005-01), respectively. Clathrin and AP-1 μ1A depletions have been described previously (42,46). HeLa cells were transfected with siRNA using Oligofectamine (Invitrogen), as specified by the manufacturer. For efficient knockdown, two transfections were performed 2 days apart, and experiments were carried out 2 days after the second knockdown.

### Transmission electron microscopy

#### Resin embedding of whole cells

For routine EM, auxilin 1/auxilin 2-depleted cells, dynamin 2-depleted cells and control cells were fixed in tissue culture dishes by adding double-strength fixative [5% glutaraldehyde (GA) and 4% PFA in 0.2 m sodium cacodylate, pH 7.3] to an equal volume of tissue culture medium for 2 min at 37°C. The fixative was then replaced by 2.5% GA and 2% PFA in 0.1 m sodium cacodylate buffer, pH 7.3. The cells were fixed for a further hour at room temperature, washed with 0.1 m sodium cacodylate buffer, pH 7.3, and pelleted at 10 000 × ***g*** for 5 min. The cell pellets were post-fixed with 1% osmium tetroxide in 0.1 m sodium cacodylate buffer, pH 7.3, for 1 h, washed with 0.05 m sodium maleate buffer, pH 5.2. They were then either *en bloc* stained with 0.5% uranyl acetate in 0.05 m sodium maleate buffer for 1 h or impregnated with 1% tannic acid in 0.1 m sodium cacodylate buffer, pH 7.3, for 2 h. The cell pellets were dehydrated in ethanol, exchanged into 1,2-epoxy propane and embedded in Araldite CY212 epoxy resin (Agar Scientific). Ultrathin sections were collected onto formvar/carbon-coated EM grids and stained with uranyl acetate and Reynolds lead citrate. The sections were observed in a transmission electron microscope (model CM 100; Philips) at 80 kV.

For quantitative analysis of clathrin-coated structures in the cytoplasm of control and auxilin 1/auxilin 2-depleted HeLa cells, cells were pre-permeabilized with 0.05% saponin in cytosol buffer for 30 seconds, quickly washed in cytosol buffer and immediately fixed in 2.5% GA and 2% PFA in 0.1 m sodium cacodylate buffer, pH 7.3, and processed as described above. The cytoplasm of individual cell profiles was photographed at a magnification of ×21 000, and the clathrin-coated structures were counted. For each condition, at least 60 images were analyzed from two independent experiments. In control cells, 258 clathrin-coated profiles were counted, of which 169 had a clearly visible membrane. In auxilin 1/auxilin 2-depleted HeLa cells, of 344 clathrin-coated structures, only 82 had a visible membrane. The diameter of the same clathrin-coated structures was measured using velocity software, and for each structure, two measurements were averaged.

#### Resin embedding of CCVs

For morphological analysis of isolated clathrin-coated structures, CCV pellets were immediately fixed in 2% PFA/2.5% GA in 0.1 m sodium cacodylate buffer, pH 6.5, for 1 h at room temperature and then processed as described above. Micrographs of random fields within the sectioned CCV pellet were taken, and the diameter of the clathrin-coated structures was measured using velocity software. For the control cells, 3369 structures from two separate preparations were analyzed, and for the auxilin 1/auxilin 2-depleted cells, 2534 structures from three separate preparations were analyzed.

#### Ruthenium red

To analyze clathrin-coated profiles at the plasma membrane, cells were fixed in 4% PFA/2.5% GA in 0.1 m sodium cacodylate buffer, pH 7.3, containing 0.5 mg/mL ruthenium red (Fluka) for 1 h at room temperature, washed with 0.15 m sodium cacodylate buffer, pH 7.3, and post-fixed with 1% osmium tetroxide in 0.1 m sodium cacodylate buffer containing 0.5 mg/mL ruthenium red for 3 h at room temperature. Cells were washed with 0.15 m sodium cacodylate buffer, pH 7.3, scraped off the dish in 0.15 m cacodylate buffer and spun for 1 min at 800 × ***g*** in a benchtop centrifuge. The cell pellets were post-fixed with osmium tetroxide, dehydrated and embedded as described above. Quantitative analysis of the percentage of plasma membrane occupied by clathrin-coated profiles was determined by applying standard stereology methods (48,49). Grids were systematically scanned at a magnification of ×39 000, and more than 240 images for each condition were captured using a digital camera (Megaview III TEM; Soft Imaging System). A total of 784 images were analyzed with adobe photoshop software, using a 500-nm lattice overlay to score intersections with the plasma membrane and clathrin-coated profiles within 200 nm of the plasma membrane. 3448 plasma membrane intersections were scored for control cells, 4605 for auxilin 1/auxilin 2-depleted cells and 3330 for dynamin 2-depleted cells. Clathrin-coated structures were grouped into three categories according to their morphology. The category of shallow and curved pits includes pits ranging from flat to ‘U’ shaped in morphology. Deeply curved pits, i.e. ‘omega’ shaped, with narrow necks, or appearing completely sealed but containing ruthenium red, were grouped into the category of deeply invaginated pits. Free endocytic CCVs were defined as ruthenium red-negative clathrin-coated structures within 200 nm of the plasma membrane.

### EM tomography

#### Data collection

CCV pellets from auxilin-depleted cells were homogenized in Buffer A [0.1 m Mes, pH 6.5, 0.2 mm EGTA, 0.5 mm MgCl_2_, 0.02% NaN_3_, 0.2 mm AEBSF (4-(-2-aminoethyl) benzenesulfonyl fluoride)] and mixed with 10 nm colloid gold particles before applying to holey carbon grids. The grids were briefly blotted with filter paper and plunged into liquid ethane slush. Tomographic tilt series were collected using a Tecnai F30 microscope (FEI), operating at 200 kV, and UCSF Tomography software. A typical data set was from −60 to +60 degrees with 2 degree increments. The nominal magnification was set at ×20 000, resulting in a final pixel size of 7.15 Å on a 2K × 2K Tietz CCD (F224HD). The defocus was set to ∼7 μm, and the final accumulative dose on the specimen was ∼50 e^−^/Å^2^.

#### Image processing

The program package imod was used to align the tomographic tilt series, and the aligned image stack was normalized using Bsoft software. The final reconstructed volume was generated using the Spider program. Volumes containing clathrin-coated structures were displayed using imod, and individual assemblies were boxed out from the 3D reconstructed tomograms and presented as a series of parallel slices. The slice thickness is 14.3 Å. Images have been contrasted in adobe photoshop.

### Auxilin quantification

The relative abundance of auxilin 1 and auxilin 2 in HeLa cell CCVs was estimated using emPAIs extracted from the proteomics data published in Borner et al. (22). emPAI values were calculated based on the method described in Ishihama et al. (50).

## Supplementary material

Figure S1 Auxilin depletion results in increased levels of transferrin receptor at the plasma membrane Cells were depleted of auxilins or dynamin and incubated with ^125^I-labeled transferrin or EGF for 30 min at 4°C. Each point is derived from at least four separate experiments performed in triplicate, and the error bars show the standard error of the mean. There is no significant change in the levels of EGF binding, but there is a twofold to threefold increase in transferrin binding in auxilin- and dynamin-depleted cells. The increased amount of transferrin receptor at the cell surface is most likely a consequence of a block in its internalization.

Figure S2 Brefeldin A sensitivity in auxilin-depleted cells Cells depleted of both auxilins were treated with 20 μg/mL Brefeldin A for 5 min at 37°C, fixed and labeled with antibodies against AP-1γ, GGA1 or GGA2. The increased labeling on intracellular membranes in auxilin-depleted cells is Brefeldin A sensitive, suggesting that in auxilin-depleted cells, the association of adaptors with intracellular membranes is still dependent on the presence of ARF1. Scale bar: 20 μm.

Figure S3 Clathrin-coated structures isolated from auxilin-depleted cells are more uniformly round than those isolated from control cells A) Ratio of short axis to long axis for clathrin-coated structures isolated from control and auxilin-depleted cells. A higher percentage of the structures from auxilin-depleted cells have ratios of 0.9–1, whereas a higher percentage of the structures from control cells have ratios of less than 0.9. B) Electron micrograph of a control cell treated with saponin before fixation. Three post-Golgi clathrin-coated profiles are indicated. The arrow points to a round clathrin-coated bud or vesicle; the arrowheads point to two elliptical buds with clathrin coating on one side only. Such buds, when sheared off, likely give rise to the irregularly shaped CCVs in our preparations. Scale bar: 250 nm.
